# Effect of tetrahedral DNA nanostructures on proliferation and osteogenic differentiation of human periodontal ligament stem cells

**DOI:** 10.1111/cpr.12566

**Published:** 2019-03-18

**Authors:** Mi Zhou, Nanxin Liu, Qi Zhang, Taoran Tian, Quanquan Ma, Tao Zhang, Xiaoxiao Cai

**Affiliations:** ^1^ State Key Laboratory of Oral Diseases West China Hospital of Stomatology, Sichuan University Chengdu China

**Keywords:** nanomaterials, osteogenic differentiation, periodontal ligament stem cells, tetrahedral DNA nanostructure

## Abstract

**Objective:**

To explore the effects and underlying biological mechanisms of tetrahedral DNA nanostructures (TDNs) on the proliferation and osteogenic differentiation of periodontal ligament stem cells (PDLSCs).

**Materials and methods:**

Real‐time cell analysis (RTCA) and CCK8 were used to screen the best concentration of TDN for PDLSCs. Cell proliferation and osteogenic differentiation were assessed after PDLSCs were treated with TDN. Data were analysed using one‐way ANOVA.

**Results:**

Tetrahedral DNA nanostructures could play a crucial role in accelerating the proliferation of PDLSCs and had the strongest promotive effect on PDLSCs at a concentration of 250 nmol/L. Simultaneously, the osteogenic differentiation of PDLSCs could be promoted significantly by TDNs and the finding displayed that the Wnt/β‐catenin signalling pathway might be the underlying biological mechanisms of TDNs on promoting the osteogenic differentiation of PDLSCs.

**Conclusion:**

Tetrahedral DNA nanostructure treatment facilitated the proliferation of PDLSCs, significantly promoted osteogenic differentiation by regulating the Wnt/β‐catenin signalling pathway. Therefore, TDNs could be a novel nanomaterial with great potential for application to PDLSC‐based bone tissue engineering.

## 1 INTRODUCTION

As a relatively common disease, bone defects can be caused by a multitude of factors, which are mainly divided into two types: congenital factors such as cheilopalatognathus, nasofacial defect and congenital ear injury and acquired factors such as trauma, tumour, injection and surgical debridement of osteomyelitis. With the improvement in materials and technology, more and more therapies are being developed to rebuild bone defects, but the results remain unsatisfactory.^1^, ^2^, ^3^ Therefore, the means to repair tissue defects successfully are still a tricky problem. Allograft bone graft, autogenous bone graft, distraction osteogenesis and application of biomaterials are the most commonly used treatments for bone defects; however, the disadvantages that include the opening up of a second operation area, severe trauma, high immunogenicity and insufficient osteogenesis cannot be ignored.^4^, ^5^ Tissue engineering thus represents the coming of “the new age of regenerative medicine” as it is deemed as the most ideal therapeutic method for the reconstruction of bone defects in the recent decades.^6^, ^7^, ^8^, ^9^, ^10^ Periodontal ligament stem cells (PDLSCs), a type of mesenchymal stem cells derived from the periodontal ligament, have attracted widespread attention, especially in the field of bone regeneration. They have multiple advantages such as strong clonality, self‐renewal and pluripotency, and they could be induced to differentiate, under different induction conditions, into various tissues such as nervous, bone, muscle and adipose tissues.^11^, ^12^, ^13^, ^14^ For example, according to a previous study, PDLSCs could differentiate into fibroblasts, osteoblast‐like cells and cementum cell‐like cells to generate natural periodontium‐like connective tissue, osteoid tissue and cementoid tissue. The spatial arrangement and morphological structure of these tissues mentioned above resembled the natural periodontal ligament and cementum complex.^14^ In addition, unlike bone mesenchymal stem cells (BMSCs), PDLSCs can be obtained via a simple, minimally invasive procedure and have low immunogenicity.^15^, ^16^ Consequently, PDLSCs are regarded as essential and perfect seeding cells in the fields of bone regeneration, especially for the reconstruction of the alveolar bone defect.

Since the structure DNA nanotechnology was put forward by Seeman in 1982, DNA nanomaterials have both aroused wide public concern and become a research hotspot in related fields because of their programmability, controllability and good mechanical performance.^17^, ^18^, ^19^ There are various DNA nanomaterial structures, such as one‐dimensional DNA structures including linear DNA, two‐dimensional DNA structures, which self‐assemble to form planar structures such as a triangle, a pentagram and three‐dimensional DNA structures, including octahedron and dodecahedron.^20^, ^21^, ^22^, ^23^, ^24^ However, one of the simplest and strongest three‐dimensional structural models, the tetrahedral DNA nanostructure (TDN) self‐assembled by four specific single‐stranded DNA (ssDNA) by complementary base pairing, has received the most extensive attention and application among all DNA structures. Therefore, TDN has great potential for application in molecular diagnosis, drug delivery and bioimaging. In addition, because of the merits of excellent biocompatibility, high stability, low toxicity, fast degradation and direct cellular uptake through endocytosis without carriers, TDNs have been applied to tissue engineering in order to modulate the biological behaviours of stem cells, including proliferation, migration and differentiation.^25^, ^26^, ^27^, ^28^, ^29^, ^30^, ^31^ Furthermore, Zhang et al^32^ demonstrated that by inhibiting MAPK phosphorylation, TDNs exerted an anti‐inflammatory effect on RAW264.7 cells. However, there are no studies that have applied TDNs to PDLSCs.

Considering all of this evidence, a hypothesis was proposed that TDNs may facilitate proliferation and promote osteogenic differentiation of PDLSCs by regulating the Wnt/β‐catenin signalling pathway. Therefore, PDLSCs were exposed to TDNs, with the purpose of systematically exploring the role of TDNs in the proliferation and osteogenic differentiation of PDLSCs. The results indicated that TDNs had a tremendous potential for application in PDLSC‐based bone regeneration.

## 2 MATERIALS AND METHODS

### 2.1 Materials

As shown in Table 1, well‐defined ssDNAs were synthesized by TAKARA (Dalian, China). LPS and alkaline phosphatase (ALP) assay kit, which were used to induce the inflammatory environment, were obtained from Beyotime (Shanghai, China). Osteogenic differentiation medium was purchased from Cyagen (Shanghai, China). Primary antibodies and secondary antibodies were purchased separately from Abcam (Cambridge, UK) and Signalway Antibody (Shanghai, China).

**Table 1:** Base sequence of each single‐stranded DNA

ssDNA	Direction	Sequence
S1	5′‐3′	ATTTATCACCCGCCATAGTAGACGTATCACCAGGCAGTTGAGACGAACATTCCTAAGTCTGAA
S2	5′‐3′	ACATGCGAGGGTCCAATACCGACGATTACAGCTTGCTACACGATTCAGACTTAGGAATGTTCG
S3	5′‐3′	ACTACTATGGCGGGTGATAAAACGTGTAGCAAGCTGTAATCGACGGGAAGAGCATGCCCATCC
S4	5′‐3′	ACGGTATTGGACCCTCGCATGACTCAACTGCCTGGTGATACGAGGATGGGCATGCTCTTCCCG

### 2.2 Synthesis and characterization of TDNs

Tetrahedral DNA nanostructures were prepared as reported previously with four pre‐designed DNA single strands.^33^, ^34^ In brief, these four DNA single strands at the same ratio were mixed into the TM buffer (10 mmol/L Tris‐HCl, 50 mmol/L MgCl_2_, pH 8.0), and the mixture was heated at 95°C for 10 minutes and then incubated at 4°C for 20 minutes. The successful synthesis of TDNs needed to be corroborated using polyacrylamide gel electrophoresis (PAGE; 8%). In addition, the configuration of TDNs was displayed by using atomic force microscopy (AFM) and transmission electron microscope (TEM).

### 2.3 Sample collection and PDLSC culture

The periodontal ligament tissue samples came from the extracted third molars, healthy and with no inflammation, of adults between 18 and 25 years old. We received the informed consent of the donors, and we followed the guidelines of the ethics committee of the University of Sichuan. The isolation and culture of cells have been described in previous studies.^14^ First, a solution containing PBS and 2% penicillin/streptomycin was used to flush the periodontal membrane until it turned white. Then a sterile blade was applied to scrape gently the periodontal tissue 1/3 into the root of the tooth. After that, these tissues were cut into pieces and put into a collagenase solution for 30‐40 minutes. The collagenase activity was stopped by a special medium, which included 15% foetal bovine serum (FBS), α‐MEM medium (Hyclone, Pittsburgh, PA, USA) and 500 U/mL penicillin/streptomycin solution. It was then cultured for 7‐14 days until the primary PDLSCs could be obtained. When the cells reached the second generation, the medium needed to be changed for the growth medium including α‐MEM (Hyclone), 100 U/mL penicillin/streptomycin solution and 10% FBS.

### 2.4 Cell proliferation assay

Cell counting kit‐8 (CCK‐8) and real‐time cell analysis (RTCA) were used to monitor the PDLSCs growth and choose the optimum concentration of TDNs. The PDLSCs were placed into a 16‐well plate with 5000 cells per well. After cultured with regular growth medium for 24 hours, these cells were exposed to the TDNs solutions with concentrations of 0, 125, 250 and 375 nmol/L. Then we used the CCK‐8 and RTCA to monitor cell proliferation for 24‐48 hours and choose the optimum effect concentration on the PDLSCs.

### 2.5 Flow cytometry

We used RTCA and CCK8 to analyse the effect of TDNs on PDLSC proliferation. Besides that, we also applied a cell cycle detection kit (KeyGen Biotech, Nanjing, China) to explore the effect on the cell cycle of PDLSCs. After TDN exposure for 24 hours, the PDLSCs were trypsinized by 0.25% trypsin and centrifuged at 10 300 *g* for 5 minutes. We dislodged the supernatant lightly and added the ice‐cold 70% ethyl alcohol to fix the PDLSCs overnight. The samples washed with PBS were mixed with RNase A and dyed with PI. A flow cytometer (guava easyCyte; Millipore, Billerica, MA, USA) was applied to detect the DNA contents. Finally, two computer software packages, WinMDI2.9 and WinCycle, were employed to analyse the results.

### 2.6 TDN Treatment to promote osteogenic differentiation of PDLSCs

When PDLSCs were fused to 85%, the cells were seeded with the density of 3 × 10^4^ cells/cm into a six‐well plate. Under the traditional cell culture conditions, PDLSCs were cultivated in regular α‐MEM growth medium for one day. After that, the medium was changed to the induction medium, which included l‐ascorbic acid 2‐phosphate (0.05 mmol/L), 10% FBS, β‐glycerophosphate (10 mmol/L), dexamethasone (100 nmol/L) and α‐MEM medium. Then the PDLSCs were exposed to the 250 nmol/L TDNs solution to promote the osteogenic differentiation, while the same volume of TM buffer was put into the control groups. The induction time would last for 3 and 7 days, and the medium should be replaced every 3‐4 days.

### 2.7 ALP staining

After the PDLSCs were treated with TDNs for 7 days, a BCIP/NBT ALP colour development kit (Beyotime, Shanghai, China) was applied to observe the roles played by TDNs in the osteogenic differentiation of PDLSCs. The cells were rinsed with PBS thrice to clean up residual medium. Then 4% cold paraformaldehyde was employed to fix the PDLSCs samples for 20 minutes. After washed thrice again with PBS, the cells were stained with ALP colour buffer solution for about 2 hours at 37°C until the colour of the samples became dark blue. Rinsed by PBS, the PDLSCs samples were observed with a fluorescent microscope.

### 2.8 Calcium deposition assay

The PDLSCs exposed to 250 nmol/L TDNs for 2 weeks as previously described, the calcium deposition that was considered as a late marker of the PDLSCs osteogenic differentiation was detected with Alizarin Red staining. Washed with PBS, the PDLSCs were fixed by 4% cold paraformaldehyde for 20 minutes. After that, the Alizarin Red S dye liquor (Sigma, St. Louis, MO, USA) was applied to dye the samples for a half hour at room temperature and a fluorescent microscope was used to survey the calcium deposition.

### 2.9 Quantitative reverse transcription (RT)‐PCR

For the sake of evaluating, the expression of inflammatory and osteogenic genes, such as runt‐related transcription factor 2 (*RUNX 2*), *ALP*, osteopontin (*OPN*), quantitative RT‐PCR, was employed. (Table 2) RNeasy plus mini kit (Qiagen, CA, USA) was used to isolate and purify the total RNA from PDLSCs samples treated with 250 nmol/L TDNs for 1, 3 and 7 days, respectively. According to the instructions, cDNA was obtained by using the cDNA synthesis kit (Mbi, Glen Burnie, MD, USA). Quantitative RT‐PCR was performed using a Bio‐Rad real‐time PCR system (Bio‐Rad, Hercules, CA, USA) and SYBR Green I PCR master mix. The target primers are displayed in Table 2, and the glyceraldehyde‐3‐phosphate dehydrogenase (*GAPDH*) was characterized as the housekeeping gene.

**Table 2:** Primers sequences of selected genes

Gene	Direction	Sequence
GAPDH	5′‐3′	CAGGGCTGCTTTTAACTCTGG
	3′‐5′	TGGGTGGAATCATATTGGAACA
RUNX 2	5′‐3′	TGGTTACTGTCATGGCGGGTA
	3′‐5′	TCTCAGATCGTTGAACCTGCTA
ALP	5′‐3′	ACTGGTACTCAGACAACGAGAT
	3′‐5′	ACGTCAATGTCCCTGATGTTATG
OPN	5′‐3′	GAAGTTTCGCAGACCTGACAT
	3′‐5′	GTATGCACCATTCAACTCCTCG

### 2.10 WB

Exposed to 250 nmol/L TDNs for 1, 3 and 7 days, respectively, the PDLSCs samples were rinsed with PBS softly. The cell protein extraction reagent (KeyGen Biotech) was used to harvest the total proteins. In order to make these collected proteins denature and depolymerize, the 1/4 (v/v) ratio of loading buffer was added and the mix was boiled for 4‐5 minutes. After that, the 10% SDS‐PAGE was performed to separate total albumens into different molecular weight albumens. Subsequently, the target albumens that included the osteogenic differentiation proteins, such as RUNX 2, OPN, and the Wnt signalling pathway‐related proteins, such as β‐catenin, LEF 1 and GSK‐3β, were transferred to a polyvinylidene fluoride membrane. Then the membranes containing these target proteins were immersed in the mixture solutions that included 2.5% bovine serum albumin and 2.5% skimmed milk powder for about 50 minutes to block albumens and incubated overnight with primary antibodies (Abcam) at 4°C. Next day, after rewarming for 60 minutes at 37°C, these membranes were washed thrice with TBST and incubated with secondary antibodies (Beyotime, Shanghai, China) for 1 hour. After being washed with TBST, the bands of albumens were visualized by an ECL chemiluminescence detection system (Bio‐Rad, Hercules, CA, USA). The housekeeping control protein was GAPDH.

### 2.11 Immunofluorescence staining

Periodontal ligament stem cells were seeded on the sterile 20 mm cell slides (NEST, Beijing, China) and treated with 250 nmol/L TDNs solution for 1 and 3 days. The PDLSCs samples were rinsed with PBS and fixed with 4% (w/v) cold paraformaldehyde solution. Then we used the 0.5% TritonX‐100 to permeate the cytomembrane and 5% goat serum to block the cells. The PDLSCs samples were incubated with primary antibodies (1:200 dilution, anti‐RUNX 2, anti‐OPN, anti‐β‐catenin, anti‐GSK‐3β and anti‐LEF 1) overnight at 4°C. Next day, after rewarming the samples for 30 minutes, the DyLight 488 secondary antibody (1:300 dilution) was added to incubate PDLSCs for 1 hour and DAPI was employed to stain the cell nucleus. Finally, a confocal laser microscope (A1R MP^+^; Nikon, Tokyo, Japan) was used to capture the images.

### 2.12 Statistical analysis

The result comparisons between experimental groups and control groups were obtained using the student's *t* test. The SPSS 19.0 software (IBM, Armonk, NY, USA) was applied to make statistical analysis for all the experiment data, when *P* values <0.05, they were statistically significant. All experiments were repeated at least three times.

## 3 RESULTS

### 3.1 Characterization of TDNs

**Figure 1:**
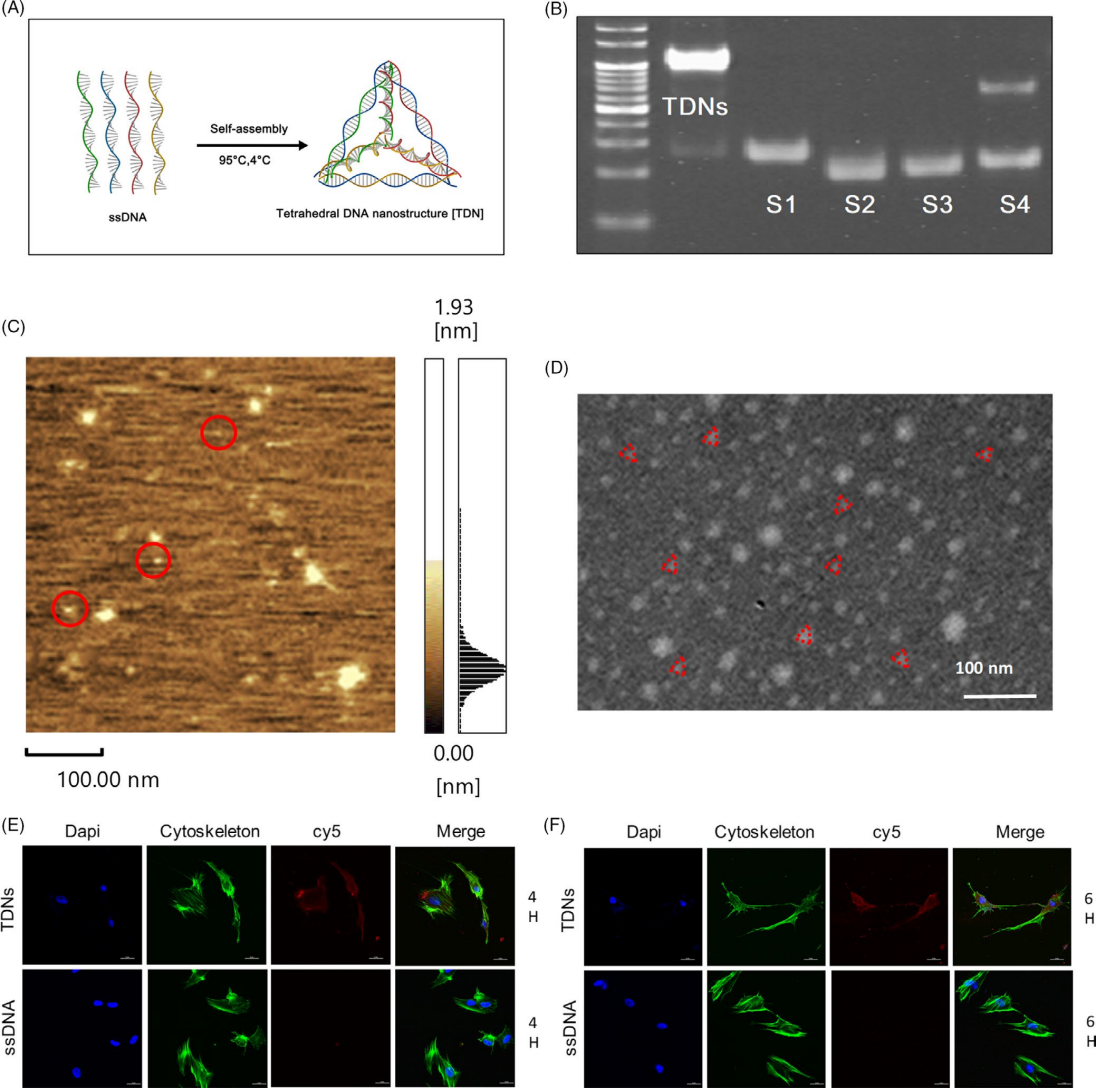
Characterization of TDNs. A, The composition diagram of TDN. B, The result of 8% PAEG. C, The scanning image of TDN by AFM. D, The scanning image of TDN by TEM. Scale bars are 100 nm. E, After treatment with TDNs for 4 h, the ability of TDN to enter PDLSCs without the assistance of vectors was analysed by immunofluorescence staining. (nucleus: blue, cytoskeleton: green, cy5: red). Scale bars are 25 µm. F, After treatment with TDNs for 6 h, the ability of TDN to enter PDLSCs without the assistance of vectors was analysed by immunofluorescence staining. (nucleus: blue, cytoskeleton: green, cy5: red). Scale bars are 25 µm.

In Figure 1A, A synthesis diagram shows the tetrahedral structure of TDNs formed by self‐folding of four pre‐synthesized ssDNA based on complementary base pairing.^25^ An 8% PAGE was performed in order to demonstrate that the tetrahedral DNA nanostructure was successfully synthesized (Figure 1B). Furthermore, we used AFM to explore the shape of TDNs, which displayed that it had a triangular particle, diverse in size, about 2‐3 nm in width and about 17 nm in height (Figure 1C).^35^ Figure 1D shows a TEM image that presents the configuration of TDNs, too. In addition, we performed immunofluorescence staining to detect the red fluorescence of CY5 tagged to S1, which proved that TDNs could enter the PDLSCs without the carrier, but a single strand of DNA could not (Figure 1E,F).

### 3.2 Effect of TDNs on proliferation

**Figure 2:**
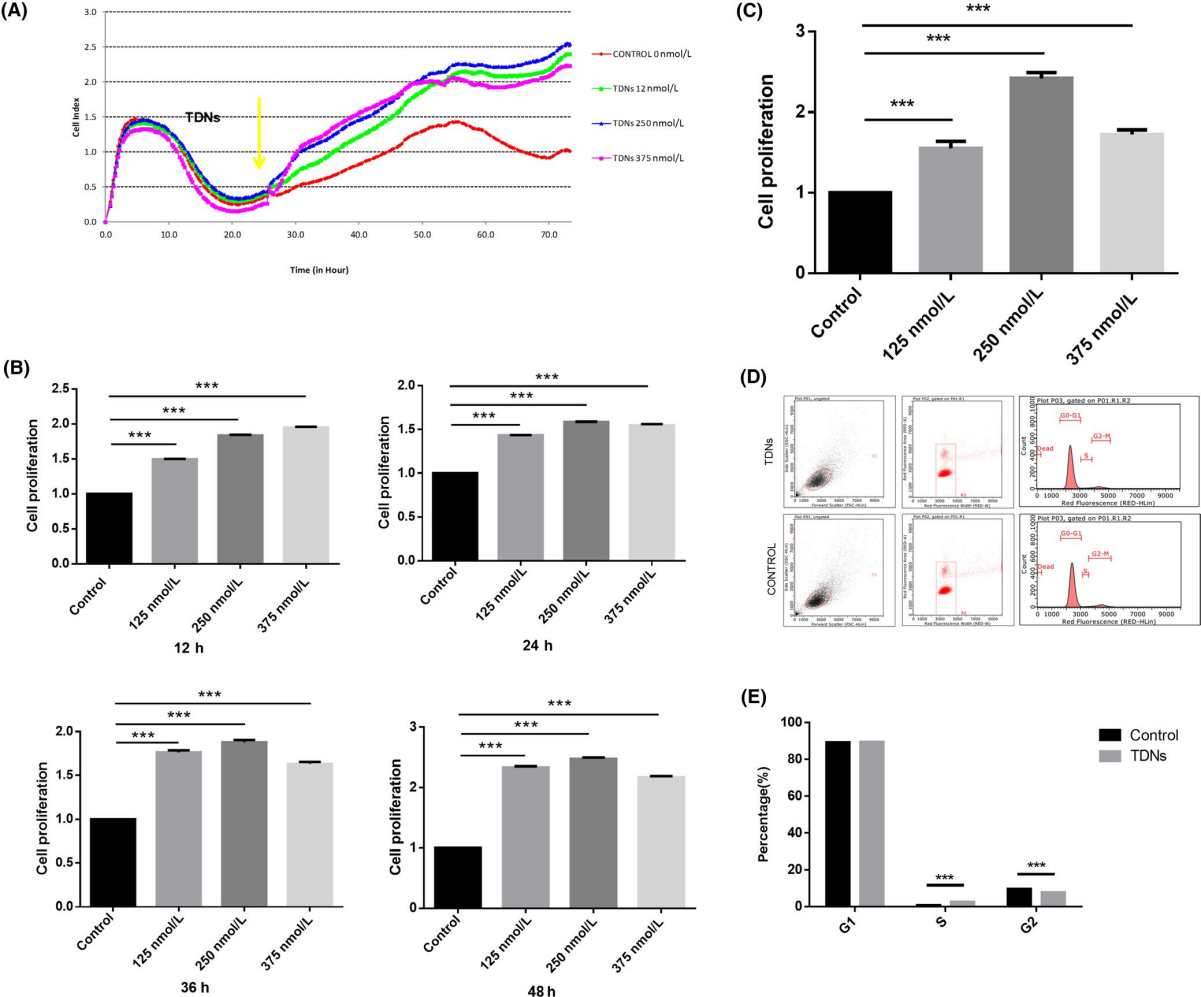
Effect of TDNs on proliferation. A, The proliferation of PDLSCs was observed by RTCA. B, Statistical analysis of RTCA results in 12, 24, 36 and 48 h. C, Statistical analysis of CCK8 results. Data are presented as means ±standard deviations (n = 3). ***P < 0.001. D, Flow cytometry was used to explore the role of TDNs on the PDLSCs by detecting cell cycle changes. E, Statistical analysis of cell cycle distribution. Data are presented as means ±standard deviations (n = 3). ***P < 0.001.

For the sake of exploring the influence of TDNs and choosing the optimum effective concentration for PDLSCs, a RTCA was performed. As shown in Figure 2A, TDNs were added as indicated by the yellow arrow. After 48 hours, the PDLSC proliferation in the experimental groups treated with TDNs was significantly higher than in the control group; when the concentration of TDNs reached 250 nmol/L, the proliferation of PDLSCs was evident (Figure 2A,B). Furthermore, we used a Cell Counting Kit‐8 to confirm the validity of the results shown above. The results of the CCK8 assay were consistent with the results of the RTCA, which demonstrated that TDNs had a significant effect on the proliferation of PDLSCs, and the optimum effect concentration was indeed 250 nmol/L (Figure 2C).

### 3.3 Effect of TDNs on cell cycle of PDLSCs

We used flow cytometry to explore the role of TDNs on the PDLSCs by detecting cell cycle changes. After PDLSCs were exposed to 250 nmol/L TDNs for one day, there was a significant difference (*P* < 0.001) between the TDN‐treated group and the control group in the number of S phase and G2 phase cells. The analysis of Figures 2D,E showed, that in comparison with control group, the ratio of PDLSCs in phase S was prominently enhanced but the ratio in phase G2 was obviously reduced; this indicated that TDNs could stimulate PDLSCs to shorten cell cycle and to speed up cell proliferation. Consequently, TDNs could change the cell cycle to regulate the proliferation of PDLSCs.

### 3.4 Effect of TDNs on the expression of osteogenesis‐related genes and albumen in PDLSCs

**Figure 3:**
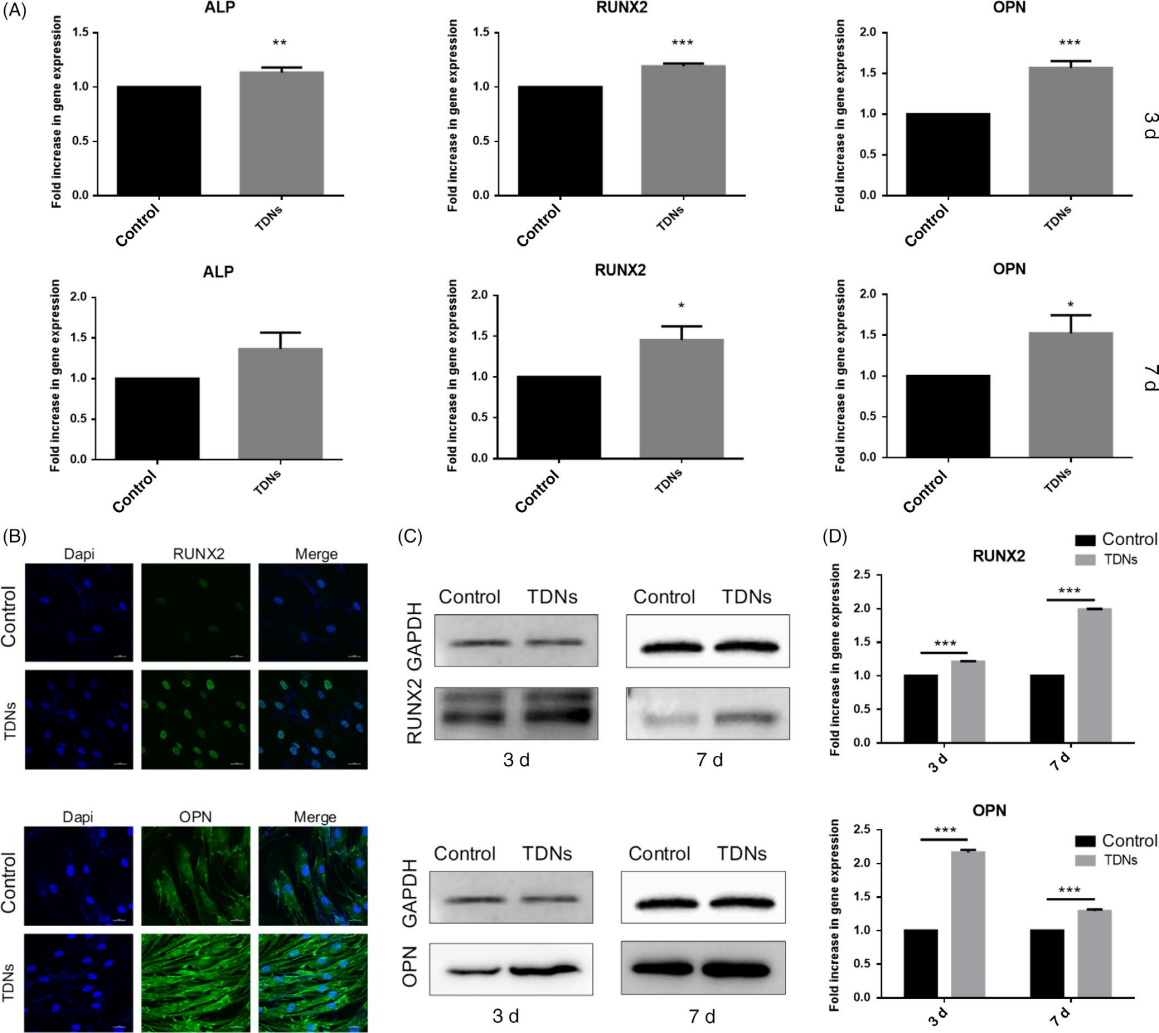
Effect of TDNs on osteogenic differentiation in PDLSCs. A, The quantitative analysis results of gene expression obtained by RT‐PCR. Data are presented as means ± standard deviations (n = 3). *P < 0.05, **P < 0.01, ***P < 0.001. B, Immunofluorescent micrographs of RUNX 2 protein and OPN protein. (nucleus: blue, RUNX 2 protein and OPN protein: green). Scale bars are 25 µm. C, The results of RUNX 2 and OPN proteins expression measured by WB. D, Statistical analysis of WB. Data are presented as means ± standard deviations (n = 3). ***P < 0.001.

As typical osteogenic‐related markers, OPN, RUNX2 and ALP were chosen to demonstrate that TDNs could facilitate the osteogenic differentiation of PDLSCs at the gene and protein levels.^36^, ^37^ Exposed to 250 nmol/L TDNs for 3 and 7 days, the gene samples of PDLSCs were analysed by RT‐PCR to characterize the part played by TDNs in the osteogenic differentiation of PDLSCs at the gene level. Quantitative analysis results were obtained by RT‐PCR (Figure 3A). The results showed the same tendency as the qualitative analysis, which manifested that TDNs had a positive effect on the osteogenic‐specific gene expression in PDLSCs.

After the gene evaluation, WB and immunofluorescence were performed to detect the albumen contents of OPN and RUNX2 regarded as osteogenic‐related markers. Following TDN treatment for 3 and 7 days, it can be seen in Figure 3C that the albumen contents of the TDN‐treated groups were up‐regulated notably. Figure 3D shows the quantitative statistical summary of the information contained in Figure 3C; it showed that RUNX2 and OPN albumen content of the TDN‐treated groups, in the 3‐day sample, was 1.21 times and 2.16 times higher than that of control group, the content of the above‐mentioned proteins was multiplied by 1.99 and 1.29, respectively, in the 7‐day samples. After being exposed to the TDNs for 3 days, OPN and RUNX2 albumens were dyed by immunofluorescence to further verification of the results. We can see that fluorescence intensity of TDN‐treated groups was clearly higher than in the control groups (Figure 3B). Overall, these results indicated that TDNs had a positive influence on the up‐regulation of osteogenic‐related genes and on the albumen expression of PDLSCs.

### 3.5 Enhancement of calcium nodule formation and ALP activity

**Figure 4:**
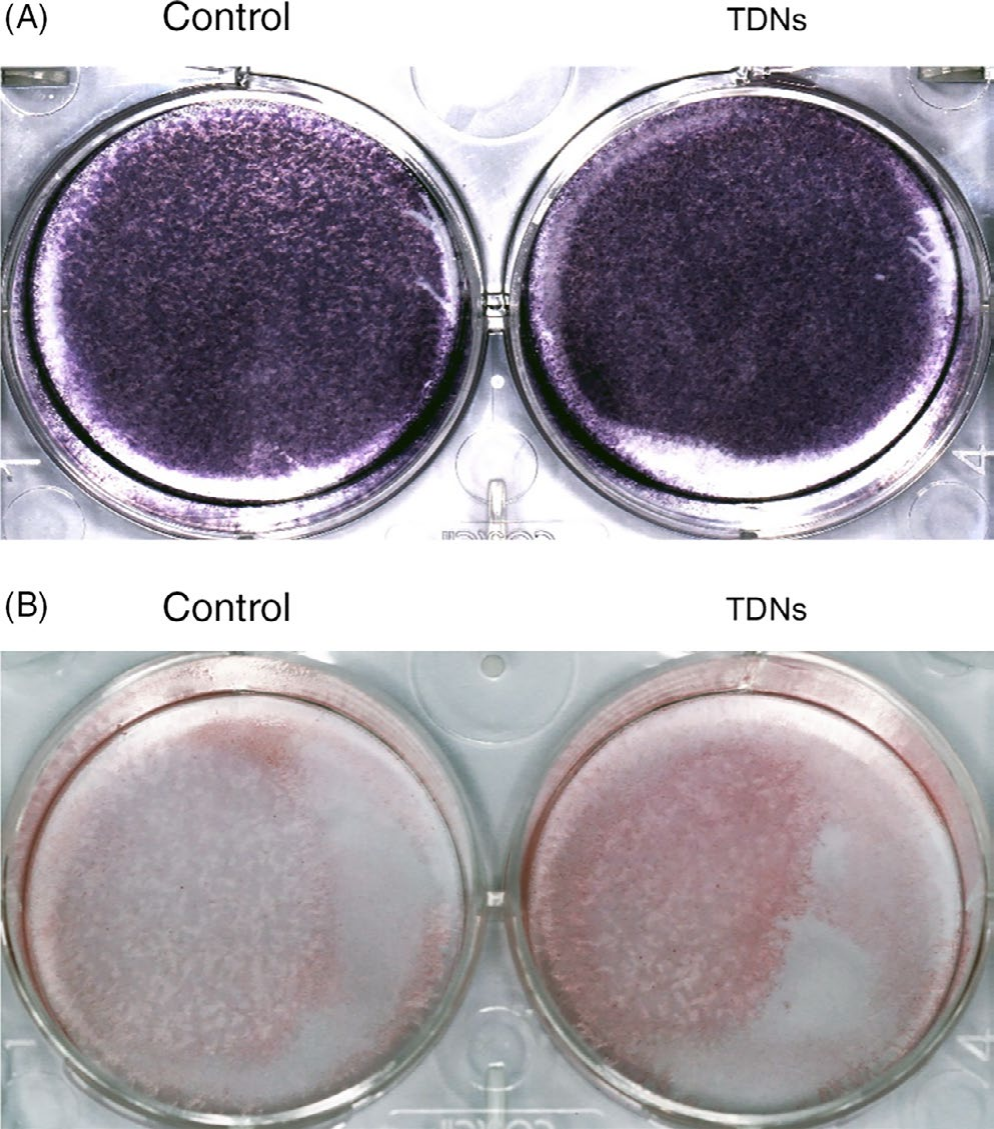
Enhancement of calcium nodule formation and ALP activity. A, Osteogenic differentiation was monitored by ALP staining at day 7. B, Osteogenic differentiation was detected by Alizarin Red staining (bottom) at day 14.

As an early marker of osteogenic differentiation, the ALP activity of PDLSCs treated with TDNs for 7 days was performed in order to evaluate the part played by TDNs on the osteogenic differentiation of PDLSCs.^38^ Compared with a blank group, there was a great enhancement of ALP activity and greater NBT formation in the TDN‐treated groups (Figure 4A).

In addition, calcium nodules, as late indicators of osteogenic differentiation, were detected by Alizarin Red staining to assess the role played by TDNs on PDLSCs.^39^ As displayed in Figure 4B, the number of calcium nodules in the experiment group exposed to TDNs for 2 weeks was remarkably higher than that in the blank group without exposure to TDNs (Figure 4B). Therefore, these results manifested that TDNs played a significant role in the PDLSCs osteogenic differentiation.

### 3.6 Mechanism through which TDN regulated the osteogenic differentiation of PDLSCs

**Figure 5:**
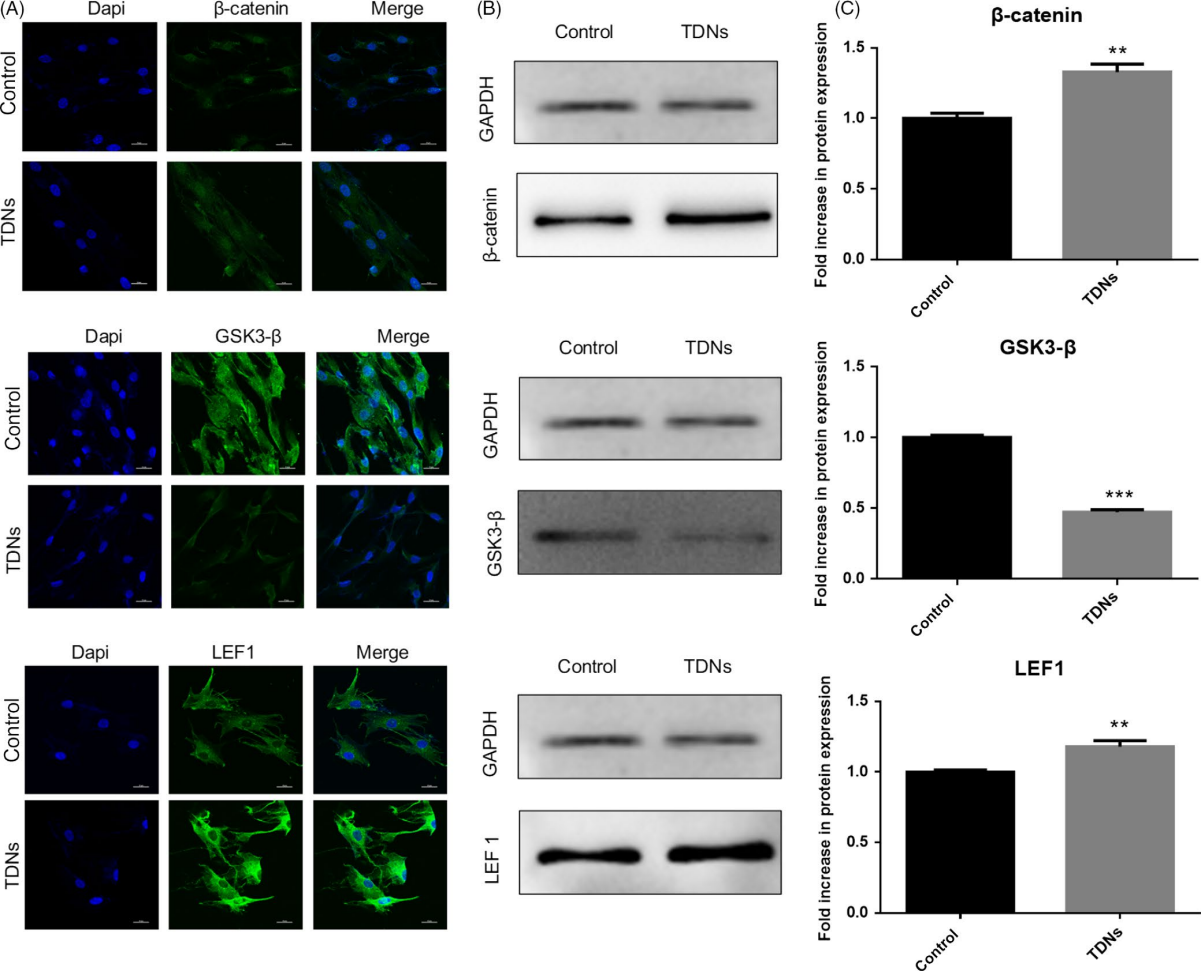
The potential mechanism through which TDN regulated the osteogenic differentiation of PDLSCs. A, Immunofluorescent micrographs of β‐catenin protein, GSK3‐β protein and LEF1 protein. (nucleus: blue, β‐catenin, GSK3‐β protein and LEF1 protein: green). Scale bars are 25 µm. B, WB analysis of the β‐catenin, GSK3‐β and LEF 1 proteins expression. C, Statistical analysis of WB. Data are presented as means ± standard deviations (n = 3). ***P < 0.001.

The mechanism through which the osteogenic differentiation of PDLSCs was increased by TDNs is still not clear. But some previous studies have demonstrated that the Wnt/β‐catenin signalling pathway plays a significant role in the osteogenic differentiation of PDLSCs.^40^, ^41^, ^42^ Therefore, in order to investigate whether the Wnt/β‐catenin signalling pathway might be the potential mechanism through which TDNs facilitate PDLSCs osteogenic differentiation, we performed western blotting (WB) and immunofluorescence staining in order to analyse the protein expression of LEF‐1, GSK‐3β and β‐catenin. Stimulated with TDNs, the WB results displayed that the expression of LEF‐1 and β‐catenin in the albumen was obviously higher than that in the control group. Simultaneously, the GSK‐3β was expressed in lower quantities in TDN‐treated group than in the control groups (Figure 5B,C). In order to reconfirm this result, we performed immunofluorescence staining, the results of immunofluorescence staining were consistent with that of WB, that is, TDNs could activate the factors related to the Wnt/β‐catenin signalling pathway (Figure 5A). Taken together, these results not only verified the previous findings but also provided new evidence, that indicated that TDNs might adjust the osteogenic differentiation of PDLSCs through activating the Wnt/β‐catenin pathway.

## 4 DISCUSSION

**Figure 6:**
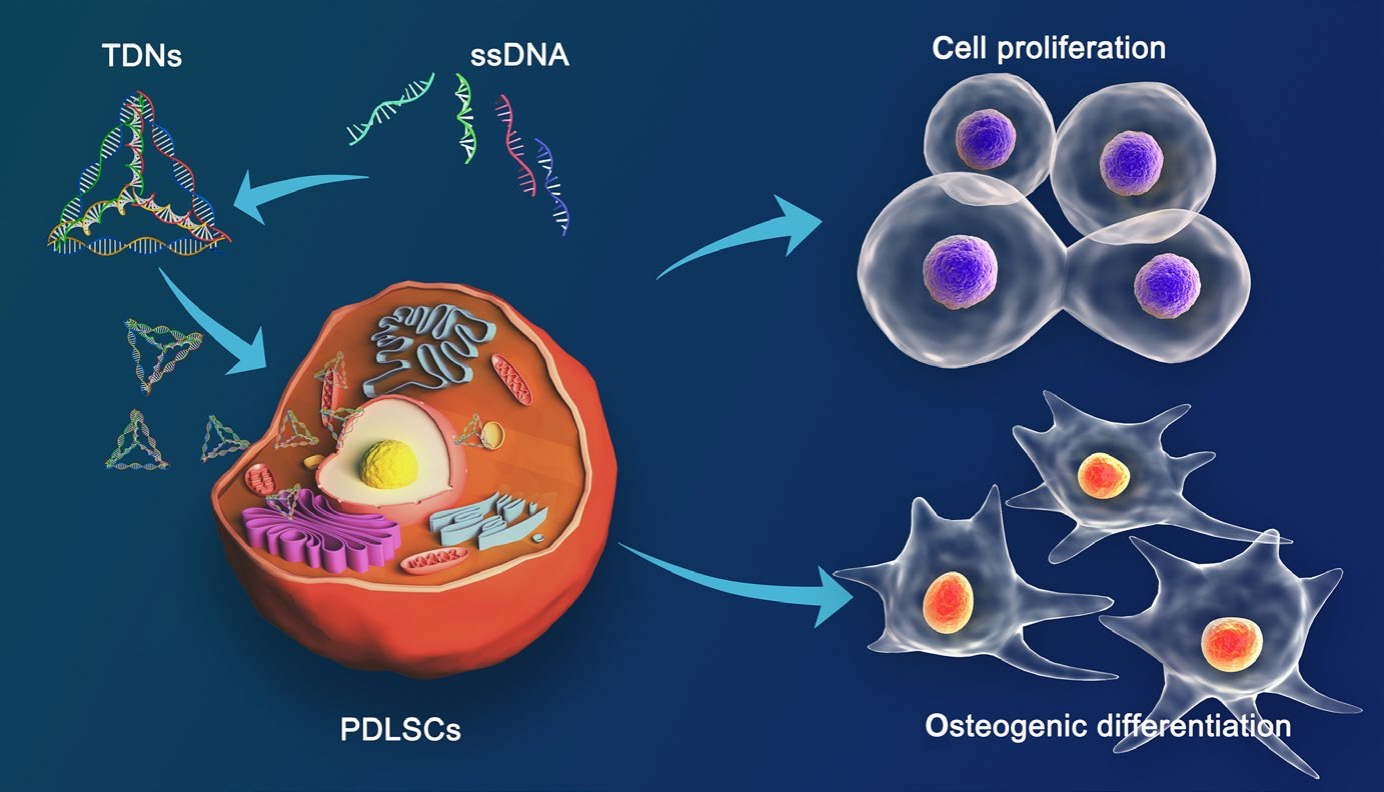
As novel DNA nanomaterials, TDNs were employed to stimulate PDLSCs to regulate its biological behaviours. The results demonstrated that TDNs could facilitate the proliferation of PDLSCs, and the optimum work concentration was 250 nmol/L. In addition, the findings also demonstrated that TDNs could greatly enhance the osteogenic differentiation of PDLSCs

As a common clinical problem, targeted repair and reconstruction of bone defects, especially alveolar bone defects, remain a challenge. Although a variety of surgical bone augmentation techniques, such as distraction osteogenesis and autogenous bone graft, have been skilfully and routinely applied to the reconstruction of bone defects, surgeons increasingly prefer bone tissue engineering due to the large trauma and high technical sensitivity required for surgical techniques. Compared with BMSCs, PDLSCs are considered as the ideal seed cells in the oral bone regeneration, owing to the advantages of small trauma and low immunogenicity. In addition, various novel biological materials are applied in PDLSC‐based tissue engineering to provide bone space for seed cells, simulate the growth microenvironment and provide nutrients or related stimulators to promote PDLSCs biological behaviours, such as proliferation and differentiation. In our experiments, TDNs, as novel DNA nanomaterials, were employed to stimulate PDLSCs to regulate its biological behaviours. The results demonstrated that TDNs could facilitate the proliferation of PDLSCs and the optimum work concentration was 250 nmol/L. In addition, the findings also demonstrated that TDNs could greatly enhance the osteogenic differentiation of PDLSCs by regulating the Wnt/β‐catenin pathway (Figure 6).

As a new three‐dimensional structure of DNA nano‐biomaterials, TDNs have garnered attention in the field of biomedicine because its structure could be functionalized and designed to respond to the stimulation of the surrounding environment. It could not only be used as a stimulator to adjust biological behaviours such as differentiation, anti‐inflammatory response, proliferation and migration of cells, but could also be applied as a biosensor in molecular diagnosis and as a special carrier to transport ligands and drugs such as siRNA, AS1411 aptamers and DOX for molecular and targeted drug delivery.^27^, ^43^, ^44^This study explored the effects of TDNs on PDLSCs for the first time, and the results suggested that it could enhance proliferation and promote osteogenesis of PDLSCs. These findings lay a firm foundation for further investigations TDNs and showed their broad application potential in the PDLSC‐based nanoengineering. In further studies, we intend to continue investigating the osteogenic differentiation effects of TDNs in animal models and to explored its synthesis with ethylene imine (PEI) to form a PEI/TDNs mixture to delay its degradation and provide sufficient for in vivo investigations.^45^ In addition, using TDNs with 3D scaffold materials to reconstruct bone defects or as a special probe to detect associated periodontal pathogens are also interesting research directions for PDLSC‐based nanoengineering.

Tetrahedral DNA nanostructure, as a new DNA nanomaterial, was applied in the PDLSC‐based nanoengineering system for the first time. The results of this investigation demonstrated that TDNs could play a crucial role in accelerating the proliferation of PDLSCs and had the strongest promotive effect on PDLSCs at a concentration of 250 nmol/L. Simultaneously, the most obvious finding of this study was that the osteogenic differentiation of PDLSCs could be promoted significantly with TDNs by regulating the Wnt/β‐catenin pathway. Therefore, TDNs could be a novel nanomaterial that could regulate some cell biological behaviours with a tremendous potential for application in PDLSC‐based bone tissue engineering, such as promoting osteogenic differentiation to restore bone defect.

## CONFLICT OF INTEREST

There is no conflict of interest.

## References

[1] Dimitriou R , Jones E , McGonagle D , Giannoudis PV . Bone regeneration: current concepts and future directions. BMC Med. 2011;9:66. 21627784 10.1186/1741-7015-9-66 PMC3123714

[2] Ashman O , Phillips AM . Treatment of non‐unions with bone defects: which option and why? Injury. 2013;44(Suppl 1):S43‐S45. 23351870 10.1016/S0020-1383(13)70010-X

[3] Giannoudis PV , Faour O , Goff T , Kanakaris N , Dimitriou R . Masquelet technique for the treatment of bone defects: tips‐tricks and future directions. Injury. 2011;42(6):591‐598. 21543068 10.1016/j.injury.2011.03.036

[4] Brydone AS , Meek D , Maclaine S . Bone grafting, orthopaedic biomaterials, and the clinical need for bone engineering. Proc Inst Mech Eng H. 2010;224(12):1329‐1343. 21287823 10.1243/09544119JEIM770

[5] Keskin D , Gundoğdu C , Atac AC . Experimental comparison of bovine‐derived xenograft, xenograft‐autologous bone marrow and autogenous bone graft for the treatment of bony defects in the rabbit ulna. Med Princ Pract. 2007;16(4):299‐305. 17541296 10.1159/000102153

[6] Cancedda R , Giannoni P , Mastrogiacomo M . A tissue engineering approach to bone repair in large animal models and in clinical practice. Biomaterials. 2007;28(29):4240‐4250. 17644173 10.1016/j.biomaterials.2007.06.023

[7] Zhang T , Lin S , Shao X , et al. Effect of matrix stiffness on osteoblast functionalization. Cell Prolif. 2017;50(3):e12338. 10.1111/cpr.12338 PMC6529113 28205330

[8] Zhang Q , Lin S , Zhang T , et al. Curved microstructures promote osteogenesis of mesenchymal stem cells via the RhoA/ROCK pathway. Cell Prolif. 2017;50(4):e12356. 10.1111/cpr.12356 PMC6529063 28714177

[9] Tian T , Liao J , Zhou T , et al. Fabrication of calcium phosphate microflowers and their extended application in bone regeneration. ACS Appl Mater Interfaces. 2017;9(36):30437‐30447. 28831802 10.1021/acsami.7b09176

[10] Liao J , Cai X , Tian T , et al. The fabrication of biomimetic biphasic CAN‐PAC hydrogel with a seamless interfacial layer applied in osteochondral defect repair. Bone Res. 2017;5:17018. 28698817 10.1038/boneres.2017.18 PMC5496380

[11] **Seo** BM , **Miura** M , **Gronthos** S , et al. Investigation of multipotent postnatal stem cells from human periodontal ligament. Lancet. 2004;364:149‐155. 15246727 10.1016/S0140-6736(04)16627-0

[12] **Singhatanadgit** W , **Donos** N , **Olsen** I . Isolation and characterization of stem cell clones from adult human ligament. Tissue Eng A. 2009;15:2625‐2636. 10.1089/ten.TEA.2008.0442 19207044

[13] Xu J , Wang W , Kapila Y , Lotz J , Kapila S . Multiple differentiation capacity of STRO‐1+/CD146+ PDL mesenchymal progenitor cells. Stem Cells Dev. 2009;18:487‐496. 18593336 10.1089/scd.2008.0113 PMC2702120

[14] **Gay** I , **Chen** S , **MacDougall** M . Isolation and characterization of multipotent human periodontal ligament stem cells. Orthod Craniofac Res. 2007;10:149‐160. 17651131 10.1111/j.1601-6343.2007.00399.x

[15] Yu BH , Zhou Q , Wang ZL . Periodontal ligament versus bone marrow mesenchymal stem cells in combination with Bio‐Oss scaffolds for ectopic and in situ bone formation: a comparative study in the rat. J Biomater Appl. 2014;29(2):243‐253. 24487130 10.1177/0885328214521846

[16] **Zhang** Q , **Shi** S , **Liu** Y , et al. Mesenchymal stem cells derived from human gingiva are capable of immunomodulatory functions and ameliorate inflammation‐related tissue destruction in experimental colitis. J Immunol. 2009;183:7787‐7798. 19923445 10.4049/jimmunol.0902318 PMC2881945

[17] Seeman NC . Nucleic acid junctions and lattices. J Theor Biol. 1982;99(2):237‐247. 6188926 10.1016/0022-5193(82)90002-9

[18] Bergamini C , Angelini P , Rhoden KJ , Porcelli AM , Fato R , Zuccheri G . A practical approach for the detection of DNA nanostructures in single live human cells by fluorescence microscopy. Methods. 2014;67(2):185‐192. 24440746 10.1016/j.ymeth.2014.01.009

[19] Li J , Pei H , Zhu B , et al. Self‐assembled multivalent DNA nanostructures for noninvasive intracellular delivery of immunostimulatory CpG oligonucleotides. ACS Nano. 2011;5(11):8783‐8789. 21988181 10.1021/nn202774x

[20] Goodman RP , Schaap IA , Tardin CF , et al. Rapid chiral assembly of rigid DNA building blocks for molecular nanofabrication. Science. 2005;310(5754):1661‐1665. 16339440 10.1126/science.1120367

[21] Rothemund PW . Folding DNA to create nanoscale shapes and patterns. Nature. 2006;440(7082):297‐302. 16541064 10.1038/nature04586

[22] Chhabra R , Sharma J , Liu Y , Rinker S , Yan H . DNA self‐assembly for nanomedicine. Adv Drug Deliv Rev. 2010;62(6):617‐625. 20230866 10.1016/j.addr.2010.03.005

[23] Shih WM , Quispe JD , Joyce GF . A 1.7‐kilobase single‐stranded DNA that folds into a nanoscale octahedron. Nature. 2004;427(6975):618‐621. 14961116 10.1038/nature02307

[24] Aldaye FA , Sleiman HF . Modular access to structurally switchable 3D discrete DNA assemblies. J Am Chem Soc. 2007;129(44):13376‐13377. 17939666 10.1021/ja075966q

[25] Shi S , Peng Q , Shao X , et al. Self‐assembled tetrahedral DNA nanostructures promote adipose‐derived stem cell migration via lncRNA XLOC 010623 and RHOA/ROCK2 signal pathway. ACS Appl Mater Interfaces. 2016;8(30):19353‐19363. 27403707 10.1021/acsami.6b06528

[26] Shi S , Lin S , Shao X , Li Q , Tao Z , Lin Y . Modulation of chondrocyte motility by tetrahedral DNA nanostructures. Cell Prolif. 2017;50(5):e12368. 10.1111/cpr.12368 PMC6529109 28792637

[27] Li Q , Zhao D , Shao X , et al. Aptamer‐modified tetrahedral DNA nanostructure for tumor‐targeted drug delivery. ACS Appl Mater Interfaces. 2017;9(42):36695‐36701. 28991436 10.1021/acsami.7b13328

[28] Shao X , Lin S , Peng Q , et al. Tetrahedral DNA nanostructure: a potential promoter for cartilage tissue regeneration via regulating chondrocyte phenotype and proliferation. Small. 2017;13(12):1602770. 10.1002/smll.201602770 28112870

[29] Shi S , Lin S , Li Y , et al. Effects of tetrahedral DNA nanostructures on autophagy in chondrocytes. Chem Commun (Camb). 2018;54:1327‐1330. 29349457 10.1039/c7cc09397g

[30] Xie X , Shao X , Ma W , et al. Overcoming drug‐resistant lung cancer by paclitaxel loaded tetrahedral DNA nanostructures. Nanoscale. 2018;10:5457‐5465. 29484330 10.1039/c7nr09692e

[31] Ma W , Shao X , Zhao D , et al. Self‐assembled tetrahedral DNA nanostructures promote neural stem cell proliferation and neuronal differentiation. ACS Appl Mater Interfaces. 2018;10(9):7892‐7900. 29424522 10.1021/acsami.8b00833

[32] Zhang Q , Lin S , Shi S , et al. Anti‐inflammatory and antioxidative effects of tetrahedral DNA nanostructures via the modulation of macrophage responses. ACS Appl Mater Interfaces. 2018;10(4):3421‐3430. 29300456 10.1021/acsami.7b17928

[33] Peng Q , Shao XR , Xie J , et al. Understanding the biomedical effects of the self‐assembled tetrahedral DNA nanostructure on living cells. ACS Appl. Mater. Interfaces. 2016;8:12733. 27153101 10.1021/acsami.6b03786

[34] Zhou M , Liu N , Shi S , et al. Effect of tetrahedral DNA nanostructures on proliferation and osteo/odontogenic differentiation of dental pulp stem cells via activation of the notch signaling pathway. Nanomed Nanotechnol Biol Med. 2018;14(4):1227-1236. 10.1016/j.nano.2018.02.004 29458214

[35] Shao XR , Lin SY , Peng Q , et al. Effect of tetrahedral DNA nanostructures on osteogenic differentiation of mesenchymal stem cells via activation of the Wnt/β‐catenin signaling pathway. Nanomedicine. 2017;13(5):1809‐1819. 28259801 10.1016/j.nano.2017.02.011

[36] Ye G , Li C , Xiang X , et al. Bone morphogenetic protein‐9 induces PDLSCs osteogenic differentiation through the ERK and p38 signal pathways. Int J Med Sci. 2014;11(10):1065‐1072. 25136261 10.7150/ijms.8473 PMC4135228

[37] Tang Y , Liu L , Wang P , Chen D , Wu Z , Tang C . Periostin promotes migration and osteogenic differentiation of human periodontal ligament mesenchymal stem cells via the Jun amino‐terminal kinases (JNK) pathway under inflammatory conditions. Cell Prolif. 2017;50(6):e12369. 10.1111/cpr.12369 PMC6529146 28833827

[38] Felthaus O , Gosau M , Morsczeck C . ZBTB16 induces osteogenic differentiation marker genes in dental follicle cells independent from RUNX2. J Periodontol. 2014;85(5):e144‐e151. 24359167 10.1902/jop.2013.130445

[39] Du L , Feng R , Ge S . PTH/SDF‐1α cotherapy promotes proliferation, migration and osteogenic differentiation of human periodontal ligament stem cells. Cell Prolif. 2016;49(5):599‐608. 27523567 10.1111/cpr.12286 PMC6496697

[40] Liu W , Konermann A , Guo T , Jäger A , Zhang L , Jin Y . Canonical Wnt signaling differently modulates osteogenic differentiation of mesenchymal stem cells derived from bone marrow and from periodontal ligament under inflammatory conditions. Biochim Biophys Acta. 2014;1840(3):1125‐1134. 24231680 10.1016/j.bbagen.2013.11.003

[41] Abd Rahman F , Mohd Ali J , Abdullah M , Abu Kasim NH , Musa S . Aspirin enhances osteogenic potential of periodontal ligament stem cells (PDLSCs) and modulates the expression profile of growth factor‐associated genes in PDLSCs. J Periodontol. 2016;87(7):837‐847. 26846966 10.1902/jop.2016.150610

[42] Jia Q , Jiang W , Ni L . Down‐regulated non‐coding RNA (lncRNA‐ANCR) promotes osteogenic differentiation of periodontal ligament stem cells. Arch Oral Biol. 2015;60(2):234‐241. 25463901 10.1016/j.archoralbio.2014.10.007

[43] Kim KR , Kim DR , Lee T , et al. Drug delivery by a self‐assembled DNA tetrahedron for overcoming drug resistance in breast cancer cells. Chem Commun (Camb). 2013;49(20):2010‐2012. 23380739 10.1039/c3cc38693g

[44] Lee H , Lytton‐Jean AK , Chen Y , et al. Molecularly self‐assembled nucleic acid nanoparticles for targeted in vivo siRNA delivery. Nat Nanotechnol. 2012;7(6):389‐393. 22659608 10.1038/nnano.2012.73 PMC3898745

[45] Tian T , Zhang T , Zhou T , Lin S , Shi S , Lin Y . Synthesis of an ethyleneimine/tetrahedral DNA nanostructure complex and its potential application as a multi‐functional delivery vehicle. Nanoscale. 2017;9:18402‐18412. 29147695 10.1039/c7nr07130b

