# CORRECTION

**DOI:** 10.1111/cpr.13543

**Published:** 2023-09-13

Correction to “Effect of tetrahedral DNA nanostructures on proliferation and osteogenic differentiation of human periodontal ligament stem cells”.

Zhou M, Liu N, Zhang Q, et al. Effect of tetrahedral DNA nanostructures on proliferation and osteogenic differentiation of human periodontal ligament stem cells. *Cell Prolif*. 2019;52:e12566. https://doi.org/10.1111/cpr.12566

In Figures 3C and 5B, the internal reference protein band of GAPDH were repeated.

**FIGURE 3:**
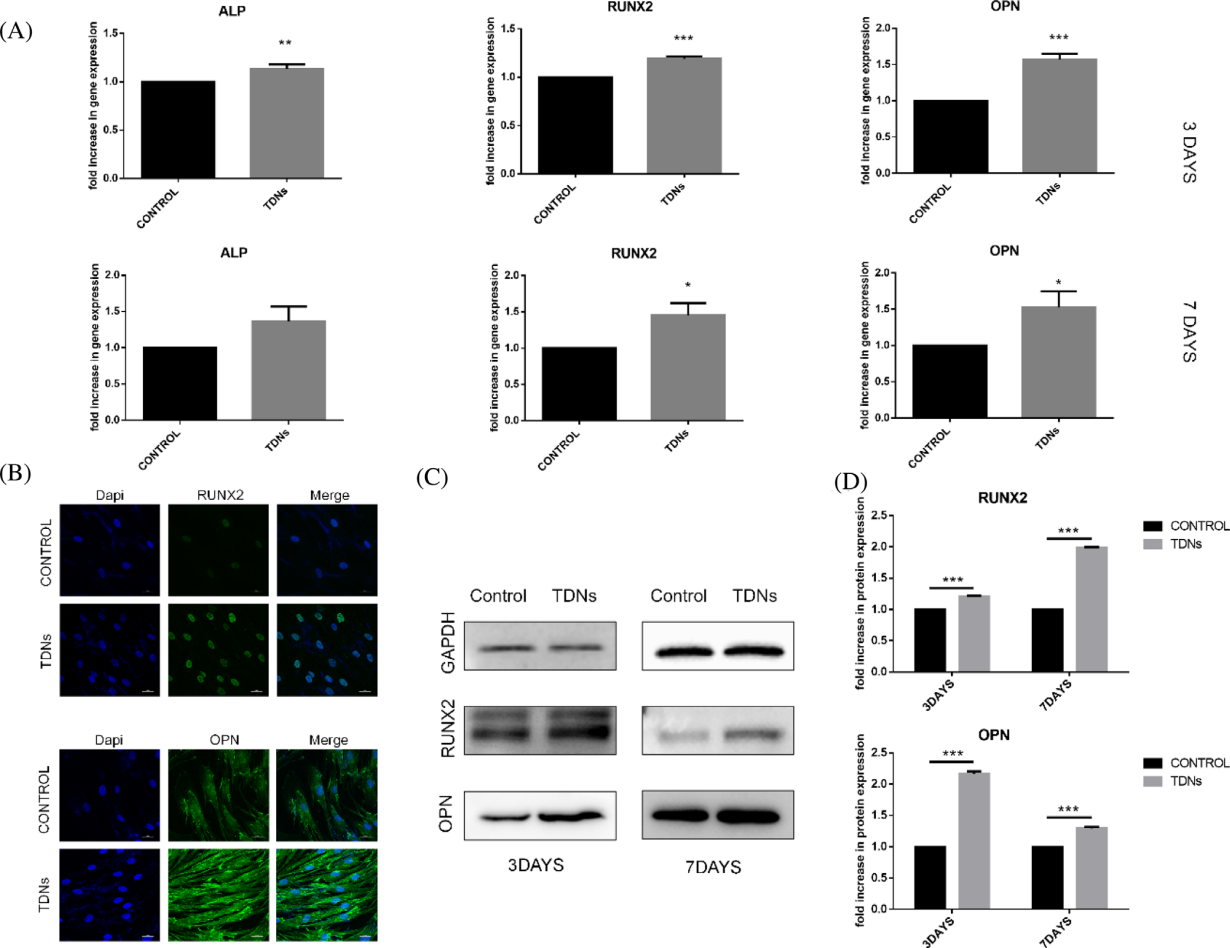


**FIGURE 5:**
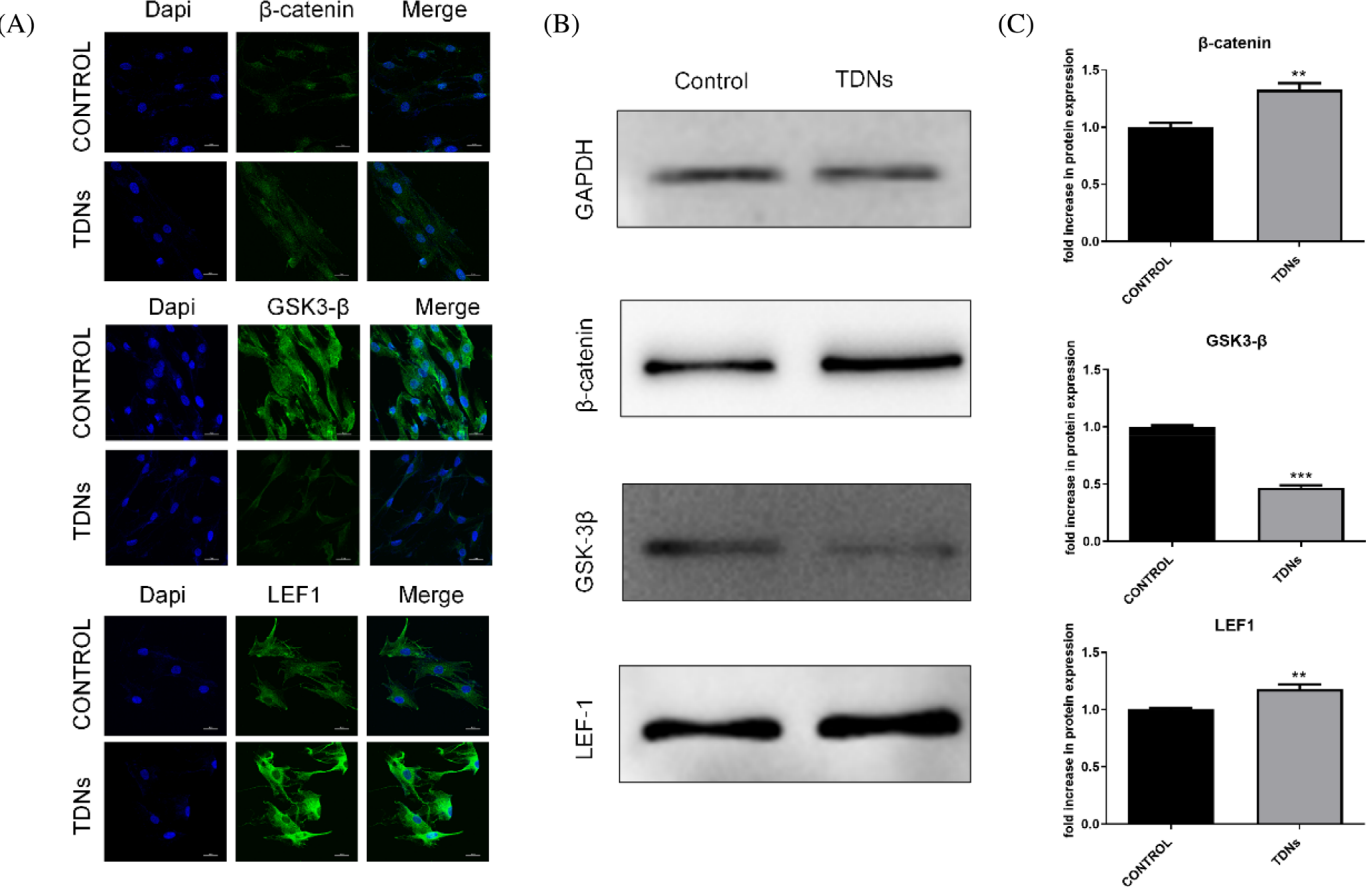


The corrected images for Figures 3 and 5 are below.

We apologise for the errors.

